# An Effective CUDA Parallelization of Projection in Iterative Tomography Reconstruction

**DOI:** 10.1371/journal.pone.0142184

**Published:** 2015-11-30

**Authors:** Lizhe Xie, Yining Hu, Bin Yan, Lin Wang, Benqiang Yang, Wenyuan Liu, Libo Zhang, Limin Luo, Huazhong Shu, Yang Chen

**Affiliations:** 1 Oral Hospital of Jiangsu Province, Affiliated to Nanjing Medical University, Jiangsu, China; 2 Centre de Recherche en Information Biomedicale Sino-Francais (LIA CRIBs), Rennes, France; 3 The Laboratory of Image Science and Technology, Southeast University, Nanjing, China; 4 The Key Laboratory of Computer Network and Information Integration (Southeast University), Ministry of Education, Beijing, China; 5 Department of Radiology, General Hospital of Shenyang Military Area Command, Shenhe District, Shenyang, China; North Shore Long Island Jewish Health System, UNITED STATES

## Abstract

Projection and back-projection are the most computationally intensive parts in Computed Tomography (CT) reconstruction, and are essential to acceleration of CT reconstruction algorithms. Compared to back-projection, parallelization efficiency in projection is highly limited by racing condition and thread unsynchronization. In this paper, a strategy of Fixed Sampling Number Projection (FSNP) is proposed to ensure the operation synchronization in the ray-driven projection with Graphical Processing Unit (GPU). Texture fetching is also used utilized to further accelerate the interpolations in both projection and back-projection. We validate the performance of this FSNP approach using both simulated and real cone-beam CT data. Experimental results show that compare to the conventional approach, the proposed FSNP method together with texture fetching is 10~16 times faster than the conventional approach based on global memory, and thus leads to more efficient iterative algorithm in CT reconstruction.

## Introduction

Computed tomography (CT) has become one of the most widely used non-invasive medical imaging systems. As the rapid development of multi-slice CT, 3-D CT has replaced the 2-D CT in radiology routines by providing fast 3-D scanning. Recently, extensive studies have demonstrated that iterative methods, based on an accurate system model, are capable of providing better reconstruction quality than analytical methods, especially under low-dose CT scans [1–10]. However, due to the high computation cost in iterative reconstructions, FBP (Filtered Back-projection) based analytical reconstructions still take the main horsepower in current clinical reconstruction for 3-D CT [11].

Projection and back-projection occupy the most computation consuming parts in CT reconstruction. How to accelerate projection and back-projection is crucial to implementing fast iterative CT reconstruction algorithms. In 2-D reconstruction, a preload system matrix can be used as projection operator, which accelerates both projection and back-projection [12]. But as to 3-D reconstruction, system matrix preload might not be feasible because the memory requirement will greatly increase. In [13], we transformed the 3-D system matrix into the combination of one single-view projection matrix and one 2-D rotation matrix, with an aim to save storage for system matrix with limited projection views [13]. However, the feasibility of this approach will be limited in the systems with large view number and large object size. For instance, in the case of a cone-beam CT system with 512 × 512 × 512 object size, 1024 × 1024 projection resolution and 400 projection views, around 8Gb memory and 4Gb memory are required to store the single sparse-view projection matrix and the corresponding rotation matrix, respectively.

GPU based parallelization has been widely used to accelerate the projection and back-projection in CT in recent years. Flores et. al parallelized the multiplication of the pre-stored system matrix by CUDA (Compute Unified Device Architecture) in 2-D iterative CT reconstruction [14]. In [15], Gao accelerated the projection and back-projection for iterative reconstructions by parallelizing the interpolation in Siddon’s ray-driven algorithm. But the thread kernel function in [15] involves complex looping and geometry parameter calculations, which often lowers the efficiency for CUDA parallelization. In [16], Zhao *et*.*al* applied GPU parallelization in projection and back-projection for iterative reconstructions, in which zero-value voxels were excluded to reduce computation cost [14]. Also, to accelerate TV (total variation) regularized iterative reconstruction, Jia *et*.*al* applied CUDA technique to accelerate ray-driven based projection and back-projection [17].

Global memory is often used in the parallelization of CT reconstruction due to its large size and direct accessibility by threads and blocks, but the applications are often compromised by its low access speed. Shared memory and texture memory both allow higher access speed, and can be considered to further enhance the parallelization efficiency. Shared memory can be directly accessed by CUDA cores, and is often used to accelerate atomic operations with sum reduction [18]. But the shared memory can only be accessed in single block, and is often too small to store the whole object data. Texture memory, though with a lower access speed than shared memory, provides faster access speed than global memory and allows efficient access to floating coordinates via texture fetching operation. Besides, the large-sized texture memory in GPU device can handle projections and scanning object, thus avoiding the frequent data exchange. Pratx *et*.*al* proposed in [19] a GPU-based (using OpenGL) 3-D OSEM (ordered subsets expectation maximization) algorithm for tomographic reconstruction in PET (positron emission tomography), in which texture memory was used to store projection data. In [20], Okistu *et*.*al* developed a CUDA framework to parallelize FDK (Feld-Davis-Kress) algorithm for CT, in which texture fetching was directly applied to accelerate the interpolation in back-projection from texture data. Also, FDK algorithm via texture fetching based parallelization was also introduced in Noël’s and Wang’s work in [21] and [22]. However, up to now, there is still no agreement on whether texture memory should be used in the parallelization of iterative reconstructions. The reason is that the texture memory is read-only, therefore the costly texture rebinding is required for data update in each iteration.

In this paper, a strategy termed Parallelization with Fixed Sampling Number Projection (FSNP) is devised to ensure the operation synchronization along projection lines in parallelizing ray-driven projection based on CUDA framework. The conventional cubic field of view (FOV) is replaced by a rotational symmetric FOV to save computation cost in geometry parameter calculation. The rest of this paper is organized as follows. In Section 2, previous works related to this study are reviewed. The proposed FSNP scheme is explained in detail in section 3. The parallelization to normalized voxel-based back-projection operator is also discussed. In Section 4, the proposed approach is applied to a cone-beam CT system Performances of different parallelization modes were compared in this section. Experiment results show that, with well synchronized operation, the FSNP method is superior in terms of computation efficiency to the conventional sampling strategy with fixed sample intervals, and the utilization of texture memory leads to further improved acceleration performance. This study also shows that the FSNP method works well with voxel-driven back-projection operator to give efficient iterative reconstruction. Table 1 lists the abbreviations used in this paper.

**Table 1:** Abbreviations.

FSNP	Fixed Sampling Number Projection
FSIP	Fixed Sampling Interval Projection
PRPT	per ray per thread
PRPB	per ray per block
CUDA	Compute Unified Device Architecture
FDK	Feld-Davis-Kress
OSEM	Ordered Subsets Expectation-Maximization
RMSE	Root Mean Square Error
SOD	Source to Orientation Distance
SDD	Source to Detector Distance

## Method

Existing algorithms for projection and back-projection can be roughly classified into three modes: ray-driven, voxel -driven and distance-driven. The ray-driven algorithm is well suited for projection and the voxel-driven algorithm works well in back-projection. However, the ray-driven back-projection tends to bring grid artifacts into image domain whereas voxel-driven projection brings grid artifacts to the projections [23]. With a more accurate geometric modeling, the distance-driven methods often lead to better performance than ray-driven projection and voxel-driven back-projection by mapping voxel and detector boundaries into the same axis [24]. Nevertheless, parallelization of the distance-driven method is highly limited by the intensive calculation of intersection areas along each ray.

Although ray-driven projector and pixel-driven back-projector are intrinsically unmatched, Zeng pointed out in [25] that unmatched projection/back-projection pair is sometimes beneficial to reconstructions, for example, a matched projection/back-projection pair with weighted line lengths often produces ring artifacts, whereas an unmatched pair, in which the projector is ray-driven with line-length-weighting but the back-projector is voxel-driven with bilinear interpolation, can effectively remove ring artifacts. Based on this, in this study we choose ray-driven projection in implementing the proposed FSNP strategy, and perform reconstructions by combining it with voxel-driven back-projection operator.

### 2.1 Projection

**Fig 1:**
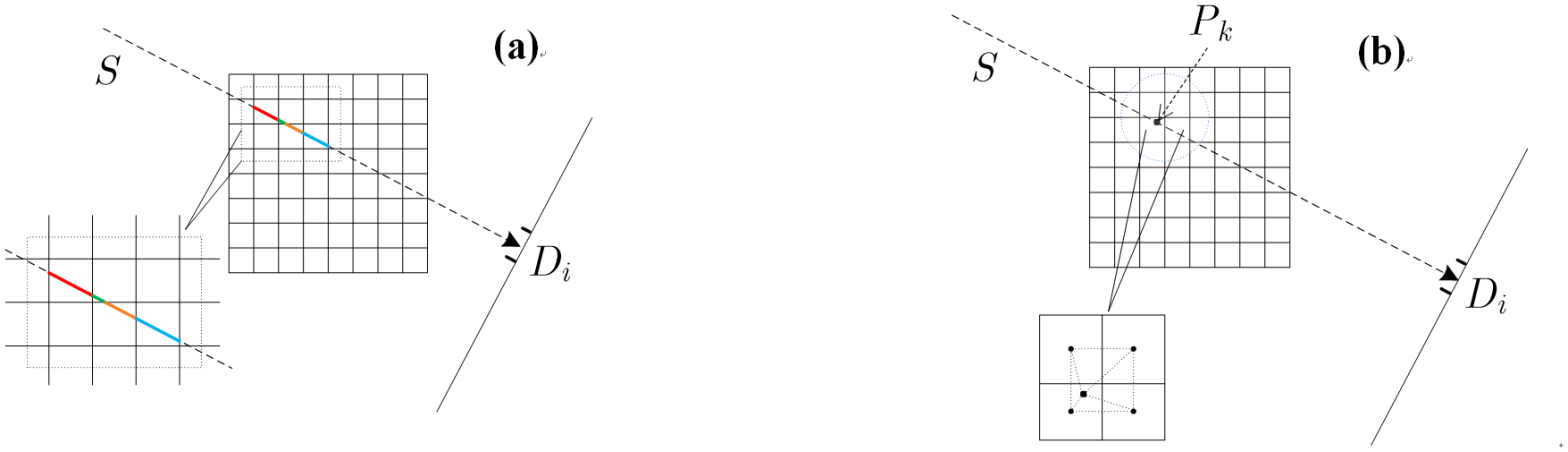
2-D illustration of ray-driven projection. (a): line summation mode; (b): linear interpolation mode.

As illustrated in Fig 1, the ray-driven algorithm works by tracing rays through scanning objects. Each ray can be simplified to an ideal line connecting the source and the detector center. Projection values are obtained via integrating sampled values along each projection ray, which is realized by a weighted intensity summation of the voxel intensities in each ray.

Different methods have been proposed to calculate the weights for voxels in projection. For instance, one can use intersection length between line and voxels [26–27], or the interpolation from neighboring voxels [27]. The classic intersection length summation based model is given in Fig 1(a). In this mode, the ideal line indexed *i* passes through pixel *j* with intensity *x*_*j*_. With the intersection length of line *i* and pixel *j* denoted by *l*_*ij*_, the contribution of pixel *j* to the projection and the summed projection value can be quantified by *x*_*j*_×*l*_*ij*_ and the projection value *y*_*i*_ = ∑_*j*_ *x*_*j*_×*l*_*ij*_, respectively. To alleviate the intensive calculation for each intersection length *l*_*ij*_ in this projection model, the line summation based model are often approximated by the linear interpolation model given in Fig 1(b). In this model, pixels surrounding each sample point in the ray are used to estimate the interpolated value $C_{p_{k}}$ of point *p*_*k*_, with weights determined by their spatial distances to the sample point. The projection value amounts to $y_{i}=\sum_{k}C_{p_{{}_{k}}}$ for detector *i*. The outline of ray-driven based projection algorithm is given below.

**Ray-driven based projection algorithm**

Set point source position *S*;

Set sampling step length *d*;

Set number of detectors *I*;

For each detector *D*_*i*_ (1≤*i*≤I)

 Compute the line equation for $\vec{SD_{i}}$, and note the unit direction vector as $\vec{n_{i}}$;

 Compute the sampling point number *m*_*i*_ for *D*_*i*_;

End For;

For each detector *D*_*i*_(1≤*i*≤I)

 Set first sampling point *P*_0_ to *S*, and set *k* to 0;

 Set projection value *y*_*i*_ to 0;

 Do

$P_{k+1}=P_{k}+\vec{n_{i}}$;

$y_{i}=y_{i}+C_{p_{k}}$;

 While $\left|\vec{SP_{k+1}}|\;<\;|\vec{SD_{i}}\right|$

End For

In ray-driven model, each projection value is calculated by integrating the sample intensities along each projection ray. Calculation of all the ray-driven projection values can be accelerated via CUDA based parallelization. Generally, the parallelization of ray-driven projection includes two options: per ray per thread (PRPT) mode and per ray per block (PRPB) mode. In PRPT mode, each projection value is independently calculated inside one single thread, which allows easy and straight parallelization. But the acceleration efficiency of PRPT mode tends to be lowered by the looping integration in each kernel function. As to the PRPB mode, each projection value is calculated within one block, and each thread in the block calculates the value for one sampling point along this projection ray. The intensive atomic operations for the intensity summing operation in PRPB mode can be effectively accelerated by sum reduction with the shared memory. So, it is generally believed that the PRPB mode is more efficient than the PRPT mode in projection parallelization [28].

**Fig 2:**
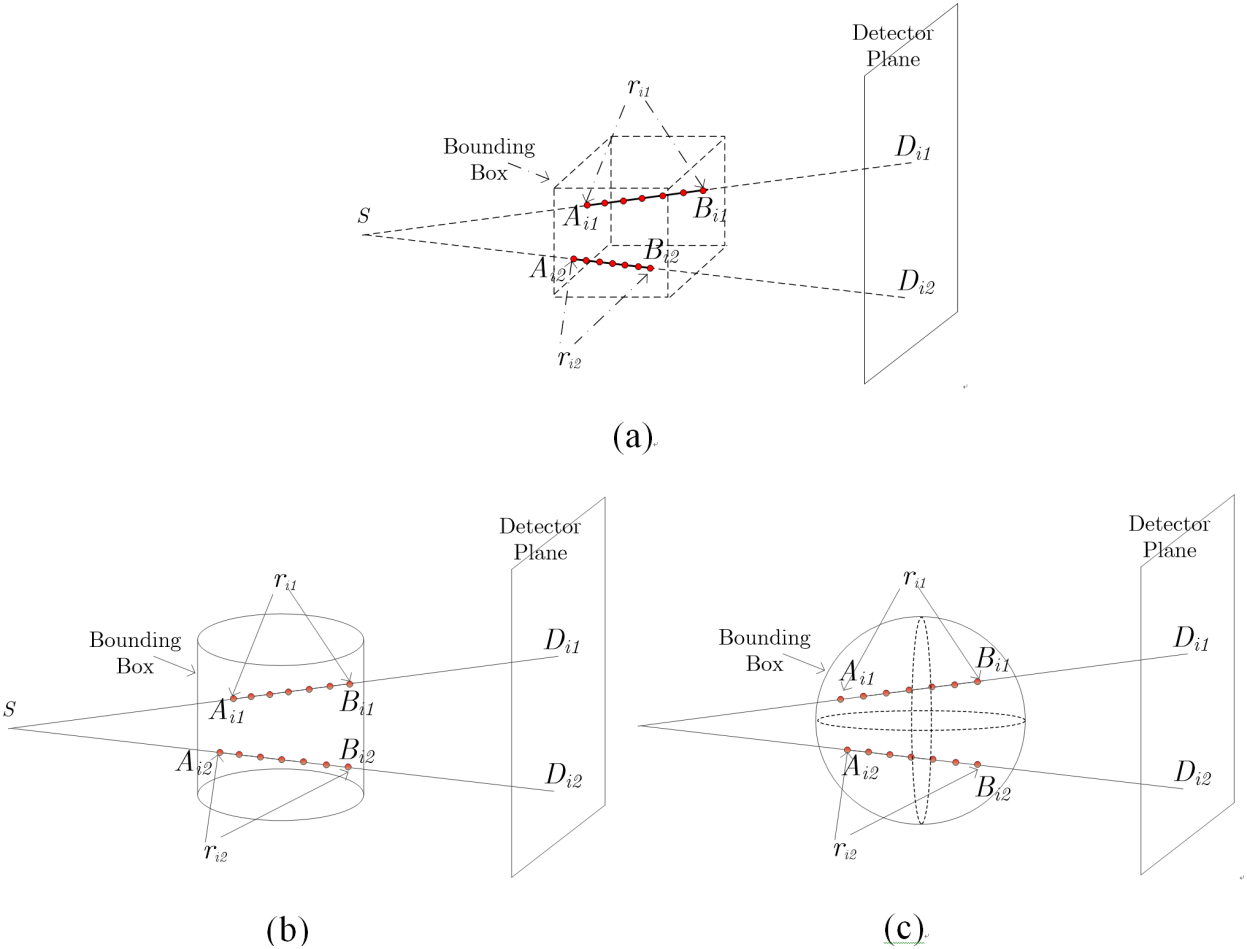
Non-uniform distributed samples points in projection geometry. (a) cubic FOV, (b) cylinder FOV, (c) sphere FOV

The sampling point numbers along different projection lines are not identical due to the varying intersection lengths for different projection lines. The varying sampling numbers will lead to unsynchronized operations in threads or blocks for PRPT or PRPB modes, which result in lowered parallelization efficiency. In this paper, a method of Fixed Sampling Number Projection (FSNP) is proposed to overcome this. As illustrated by Fig 2, this FSNP approach assumes that the X-ray attenuation is negligible outside a pre-specified field of view (FOV). We fix the sampling number along each ray and perform uniform sampling on the line segments inside the FOV, thus allows an easy synchronization for both the PRPT and PRPB modes.

In this FSNP strategy, to save computation cost, the parameters for each projection line (including intersection length, coordinates of intersection points and line direction) should be calculated and stored in memory before the projection operation. However, when the conventional cubic FOV (shown in Fig 2(a)) is used, we have to store these parameters for each projection view respectively. For a CT system with 512×512 detector plane and 360 angles, it takes about 2.5Gb memory to store the parameters for all projection views, which imposes a large saving burden for many GPU devices. To solve this, we define the FOV in cylinder and sphere shapes (as shown in Fig 2(b) and 2(c)) for long object and short object scans, respectively. In this way, for one specific detector, the intersection length keeps the same for different views, and the coordinates of intersection points and line direction can be easily calculated according to the rotational symmetry of FOV. Hence we only have to store the parameters for one view. Furthermore, in order to avoid unnecessary computation on those rays with too short intersections with the FOV, only the rays with longer intersection (with the FOV length) than one preset threshold *r*_*T*_ are considered. Normally the FOV should be large enough to ensure that no object data is removed by excluding those rays with small intersection lengths. The pseudo code for the proposed FSNP algorithm is given as follows for both the PRPT and PRPM modes:

**The proposed FSNP algorithm with PRPT mode**

//**Pre-computation part: set FOV and calculate the intersection information for start view**

Set the point source position S;

Set the sampling number on each projection line to M;

Set the field of view as FOV;

Set the detector number *I* = *D*_*row*_×*D*_*col*_; //*D*_*row*_ and *D*_*col*_ are row and column indexes of detector plane;

Set threshold *r*_*T*_ for intersection length;

For each detector *D*_*i*_ (1≤*i*≤I), do

 Compute the line equation for $\vec{SD_{i}}$;

 Compute the intersection points (*A*_*i*_, *B*_*i*_) between $\vec{SD_{i}}$ and the FOV;

 Compute *r*_*i*_ = |*A*_*i*_ *B*_*i*_|

 If (*r*_*i*_>*r*_*T*_)

  Set step length *d*_*i*_ = *r*_*i*_/(*M*−1);

 else

  Set step length *d*_*i*_ = 0 //this ray is not considered in the projection part;

End For

// **Projection Part: Calculate projection for each view**

Bind the 3-D object data in texture memory // If texture memory is used;

Update intersection information according to view angle using rotation symmetry;

Set block count *D*_*row*_ to the number of detector rows;

For *j* = 1 to *D*_*row*_,

 Calculate projection for each detector row in block *j*;

 Set thread count *D*_*col*_ to the number of detector columns;

 For *k* = 1 to *D*_*col*_, calculate current projection *y*_*i*_ in thread *k* (*i* = *j* × *D*_*col*_ + *k*);

  Set the current projection value *y*_*i*_ = 0;

  If (step length *d*_*i*_>0)

   For *m* = 1 to *M*

    Calculate the location of sampling points *p*_*m*_ on $\vec{A_{i}B_{i}}$;

    Calculate the value $C_{p_{m}}$ for point *p*_*m*_ using linear interpolation;

$y_{i}=y_{i}+C_{p_{m}}$;

   End For

  End If

 End For

 Thread Synchronize;

 Normalization: *y*_*i*_ = *y*_*i*_ × *r*_*i*_/*M*;

End For

**The proposed FSNP algorithm with PRPB mode**

//**Pre-computation part: set FOV and calculate the intersection information for start view**

Set the point source position S;

Set the sampling number on each projection line to M;

Set the field of view as FOV;

Set number of detectors I;

Set threshold *r*_*T*_ for intersection length;

For each detector *D*_*i*_ (1≤*i*≤*I*), do

 Compute the line equation for $\vec{SD_{i}}$;

 Compute the intersection points (*A*_*i*_, *B*_*i*_) between $\vec{SD_{i}}$ and the FOV;

 Compute *r*_*i*_ = |*A*_*i*_ *B*_*i*_|;

 If (*r*_*i*_>*r*_*T*_)

  Set step length *d*_*i*_ = *r*_*i*_/(*M*−1);

 else

  Set step length *d*_*i*_ = 0 //this ray is not considered in the projection part;

End For

// **Projection Part: Calculate projection for each view**

Bind the 3-D object data in texture memory; // If texture memory is used

Modify intersection information according to view angle using rotation symmetry;

Set block count I to detector numbers;

For *i* = 1 to *I*, calculate projection for each detector *D*_*i*_ in block *I*;

 If (step length *d*_*i*_>0)

  Set the projection value *y*_*i*_ = 0;

  Set thread count *M* for each block;

  For *m* = 1 to *M*, calculate each sampling value in thread *m*;

   Calculate the location parameters of sampling points *p*_*m*_ on $\vec{A_{i}B_{i}}$;

   Calculate the value $C_{p_{m}}$ for point *p*_*m*_ using linear interpolation;

   Atomic add (*y*_*i*_, $C_{p_{m}}$) (accelerated via shared memory with sum reduction);

  End For

 End If

 Thread Synchronize;

 Normalization: *y*_*i*_ = *y*_*i*_ × *r*_*i*_/*M*;

End For

### 2.2 Back-projection

**Fig 3:**
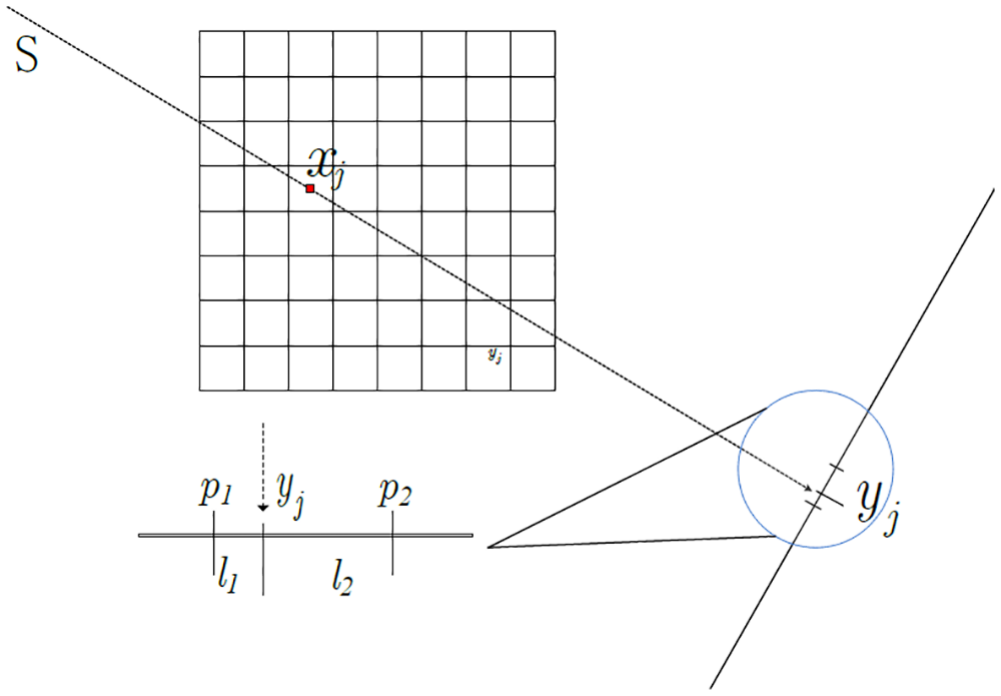
Calculation of pixel driven back-projection with linear interpolation in 2-D case.

As illustrated in the 2-D schematic diagram of the pixel-driven back-projection (Fig 3), a line (ray) connecting the radiation source and voxel center intersects with the detector plane for each pixel. Routinely, the linear interpolation or kernel function convolution can be used to estimate the back-projected value with respect to intersection locations [23–28]. Fig 3 also shows that the back-projection operator is in fact a linear interpolation: *x*_*j*_ = (*p*_1_ *l*_2_ + *p*_2_ *l*_1_)/(*l*_1_ + *l*_2_), where *l*_1_ + *l*_2_ is the distance between the two neighboring detector centers, and *p*_*1*_ and *p*_*2*_ are the corresponding projection values for the neighboring detectors. The outline of the voxel-driven based back-projection algorithm is also given below.

**Voxel-driven based back-projection algorithm**

Set point source position *S*;

Compute the coordinates of each detector center;

For *j* = 1 to *J* (*J* is the number of voxels)

 Compute the geometrical line equation that connects *S* and the voxel *j*, noted as *line*_*j*_;

 Compute the intersection point of line *line*_*j*_ and the detector array, noted as *y*_*j*_;

 Compute the back-projection value *x*_*j*_ according to the position of *y*_*j*_ via interpolation or convolution;

End For

As can be seen in above algorithm outlines, the back-projection algorithm allows a straight parallelization because the calculation related to each back-projected voxel is independent and can be easily handled by thread based parallelization. We should note that the back-projection operator in iterative reconstructions is normalized to match the projection operator by dividing the projection value by a pre-computed intersection length. The pseudo code for the parallelized back-projection is given as follows:

**Parallelized voxel-driven back-projection algorithm**

//**Pre-computation**

Set point source position *S*;

Set number of detectors *I* = *D*_*row*_ × *D*_*col*_, //*D*_*row*_ and *D*_*col*_ are row and column indexes of detector plane;

Denote *y* the projection value;

Projection normalization: ${\tilde{y}}_{i}=y_{i}/r_{i}$, // *r*_*i*_ is intersection length, computed in the projection part;

// **Calculation of the back-projected value for each voxel**

Bind the normalized 2-D projection data $\tilde{y}$ in texture memory, // If texture memory is used;

Set block number to *L* × *H*;

For p = 1 to *L* × *H* for each block

 Set thread number for each block to *W*;

 For *q* = 1 to *W*, calculate the back-projection for voxel *x*_*p*×*W*+*q*_ in each thread;

  Calculate the line equation for *l*_*p*,*q*_ that connects the S and voxel *x*_*p*×*W*+*q*_;

  Calculate the intersection point $\tilde{i}$ between the detector plane and line *l*_*p*,*q*_;

  Use linear interpolation to calculate the normalized projection on position $\tilde{i}$ as $y_{\tilde{i}}$;

  Calculate the back-projection value *x*_*p*×*W*+*q*_ from $y_{\tilde{i}}$;

 End For

 Thread Synchronize;

End For

## Experiments and Results

### 3.1 Experiment Configuration

Experiments with a simulated cone beam CT system were performed with configurations listed in Table 2. The involved abbreviations are listed in Table 2. A 3-D Shepp-Logan phantom is used. The experiments were conducted for low resolution mode and high resolution mode. In low resolution mode, the projection size is 512×512, and the scanning object size is 256×256×256; in high resolution mode, the projection size is 1024×1024, and the scanning object size is 512×512×512. We use sphere FOV shown in Fig 2(c) in the experiment. The implementation runs under a mobile workstation with Intel Core^™^ 2.40GHz (Quad-Core), 24Gb RAM, NVIDIA GTX780M graphic card (4Gb global memory), and the developing environment is Microsoft Visual Studio 2008 with CUDA version 5.5.

**Table 2:** Parameters of the simulated cone beam system.

Configuration of the simulated cone beam system	Low Resolution	High Resolution
SOD	720mm	720mm
SDD	1440mm	1440mm
Scanning Object Size	256^3^	512^3^
Object Voxel Size	0.42mm^3^	0.21mm^3^
Projection Size	512^2^	1024^2^
Projection Pixel Size	0.42mm^2^	0.21mm^2^

### 3.2 Computation Cost

We evaluated the efficiency of the proposed FSNP method for projection computation in this section. Both PRPB and PRPT modes were implemented. For each mode, both global memory and texture memory versions are considered. The computation time is listed in Table 3 in the unit of millisecond (ms). We can see in Table 3 that the PRPB mode is faster than the PRPT mode in the case when global memory is used, but the projection with PRPT mode becomes more efficient when texture memory is used for interpolation. We can also see that in global memory version, the time cost increases linearly as the sampling point number increases. But for the texture memory version, increasing sampling point number did not result in a linear increment of computation cost. The computational time increases little when sampling point number was doubled for texture based PRPT mode (around 10% for low resolution mode and 7% for high resolution mode). This means that developing projection model with higher resolution is allowed without much extra computation cost for the proposed FSNP approach when using PRPT mode with texture memory.

**Table 3:** Computational time (in ms) for the FSNP apprach with different modes

	Low Resolution		High Resolution	
	256 samples	512 samples	512 samples	1024 samples
Global PRPB	57.34	103.75	311.92	563.99
Global PRPT	117.95	219.30	1432.1	2176.4
Texture PRPB	32.65	53.59	205.12	297.28
Texture PRPT	17.09	18.95	132.29	141.31
FSIP PRPT	27.77	36.53	197.55	288.28

We also compared the computation time of the FSNP method with the conventional FSIP (Fixed Sampling Interval Projection) method in PRPT mode in Table 3. For the FSNP method, the threshold *r*_*T*_ is set to 30mm. As to the FSIP method, we adjusted the sampling density to ensure that the average sampling point number is close to the fixed sample number in FSNP method. We can see that the proposed FSNP method is faster than the FSIP method due to the better synchronization.

From Table 3, we can see that, in the texture memory version with PRPT mode, the computational time remains at same level when the sampling point number doubles for both FSNP and FSIP modes. Thus the blob based projection can be used to give better suppression of streak artifacts and artifacts [29–30]. Based on [31], the kernel function of blobs is constructed using generalized Kaiser-Bessel (KB) window *b*_*n*,*a*,*α*_:

$$b_{n,a,\alpha }(r)=\frac{1}{I_{n}(\alpha )}{\left(\sqrt{(1-{\left(r/a\right)}^{2}}\right)}^{n}I_{n}\left(\alpha \sqrt{(1-{\left(r/a\right)}^{2}}\right)\;,\;0\leq r\leq a$$ (1)

where *r* is the radial distance to the origin. *I*_*n*_ is the modified Bessel function with order m, *α* is a parameter controls the shape of the blob, *a* is the radius. Within our experiment, the parameters were set as follows: *n* = 2, *α* = 10.4, *a* = 0.84 mm (two voxels). In the current CUDA version, only nearest neighbor and linear interpolation modes are supported by texture fetching. Both modes can be applied for the computation of kernel computation. If linear interpolation mode is considered, for each sampling point, the contribution of each nearby voxels is determined by the distances between the voxel centers and the sampling points. The computation of the distances has to be implemented serially in threads with PRPT mode, which implies lowered computation efficiency. We use nearest neighbor instead. A set of discrete blob kernel functions were pre-stored in global memory (100^3^ in our experiment), each of the kernel function are with a different translation from the origin. The translation {kx,ky,kz}∈{(−12,12),(−12,12),(−12,12)}. For each sampling point, the closest blob kernel function is chosen for the interpolation. The computation cost with blob based kernel function in Table 4 indicates that the blob based projection can be accelerated by the proposed PRPT method with texture memory. Table 4 also shows that, similar to the linear interpolation based FSNP, the increment of computational time is around 14% to 30% when the sampling number is doubled for the blob based FSNP with PRPT mode.

**Table 4:** Comparison of computational time for linear interpolation based FSNP and blob based FSNP (in ms)

Sampling Points	Low Resolution		High Resolution	
	256 samples	512 samples	512 samples	1024 samples
Global PRPB Linear	57.34	103.75	311.92	563.99
Global PRPB Blob	431.92	798.15	2088.1	3808.0
Texture PRPT Linear	17.09	18.95	132.29	141.31
Texture PRPT Blob	117.55	134.96	706.42	921.35

The above result indicates that even with the costly operation of texture binding, texture memory can be used to improve efficiency in projection parallelization. Table 5 compares the computational cost for back-projection of parallelized voxel-driven algorithm using global and texture memories. Results in Table 5 confirm that the texture memory can also be used to accelerate back-projection.

**Table 5:** Computational time for back-projection operators, in ms.

Sampling Points	Low Resolution	High Resolution
Global Memory	28.91	225.94
Texture Memory	19.84	157.57

### 3.3 Projection and Reconstructions

In this section, we perform CT reconstructions with proposed FSNP. Conventional ray-driven and distance driven methods are also considered in the reconstruction for comparison. Root mean square error (RMSE) defined in Eq (2) was used to quantify the difference between the reconstructed 3-D images and original phantom 3-D images:

$$RMSE=\sqrt{\frac{{\left\|\hat{f}-f\right\|}_{2}^{2}}{{\left\|\hat{f}\right\|}_{2}^{2}}}$$ (2)

where $\hat{f}$ is the phantom image as ground truth, and *f* denotes the reconstructed image. $\left\|\hat{f}\right\|$ denotes the L_2_ norm calculation.

**Fig 4:**
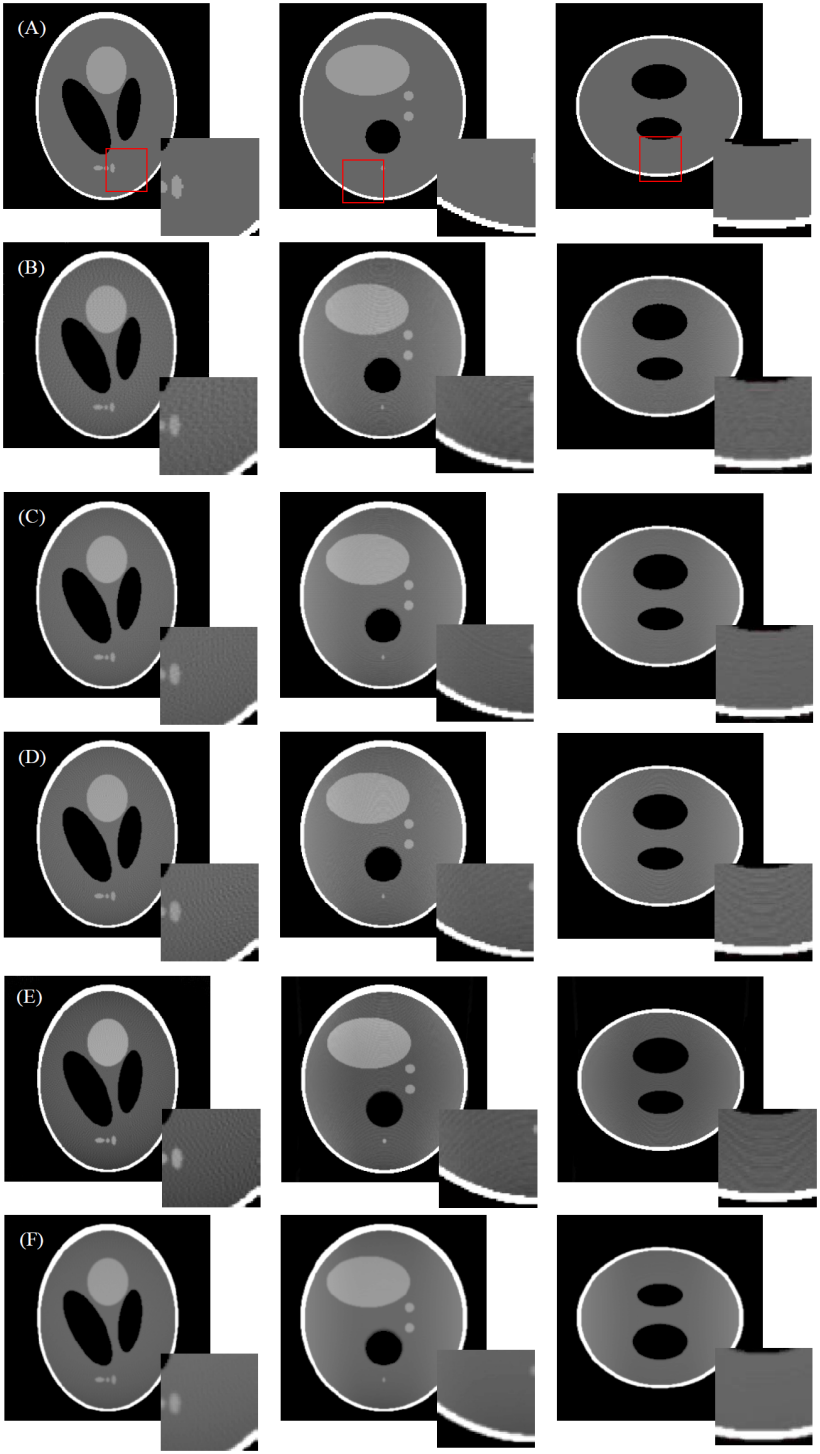
FDK reconstructions of Shepp-Logan phantom. From left to right are cross-section, sagittal view and coronal view, respectively. Row (A): phantom images; Row (B): the reconstruction with ray-driven projection and ray-driven back-projector (RMSE = 7.11%); Row (C) reconstruction with distance-driven projection and distance-driven back-projector (RMSE = 5.06%); Row (D): the reconstruction with linear interpolation based FSNP (256 sampling points per ray) and voxel-driven back-projector (RMSE = 5.30%); Row (E): the reconstruction with linear interpolation based FSNP (512 sampling points per ray) and voxel-driven back-projector (RMSE = 5.22%); Row (F): the reconstruction with blob based FSNP (256 sampling points per ray) and voxel-driven back-projector (RMSE = 2.98%).

We performed FDK reconstruction with Ram-Lak filter. 3-D Shepp-Logan phantom was used with the system configuration in Table 2 (low resolution mode), and the phantom data was projected into 360 angles. The results are shown in Fig 4. We do not provide the result for the voxel-driven projections because the grid artifacts in such projection mode often results in severe artifacts in FDK reconstruction [26]. With respect to the phantom images in Fig 4(A), obvious streak artifacts can be observed in Fig 4(B), 4(C), 4(D) and 4(E); the combination of linear interpolation based FSNP and voxel-driven back-projection operator can provide better results than the classic ray-driven projector and back-projector pair. We can also see in Fig 4(F) that the blob based FSNP leads to the reconstructions with effective artifact suppression and the lowest MSE values, but at the cost of blurred edges. This is because the simulated projections generated by blob based FSNP are blurred by the weighted summation of neighboring voxels.

**Fig 5:**
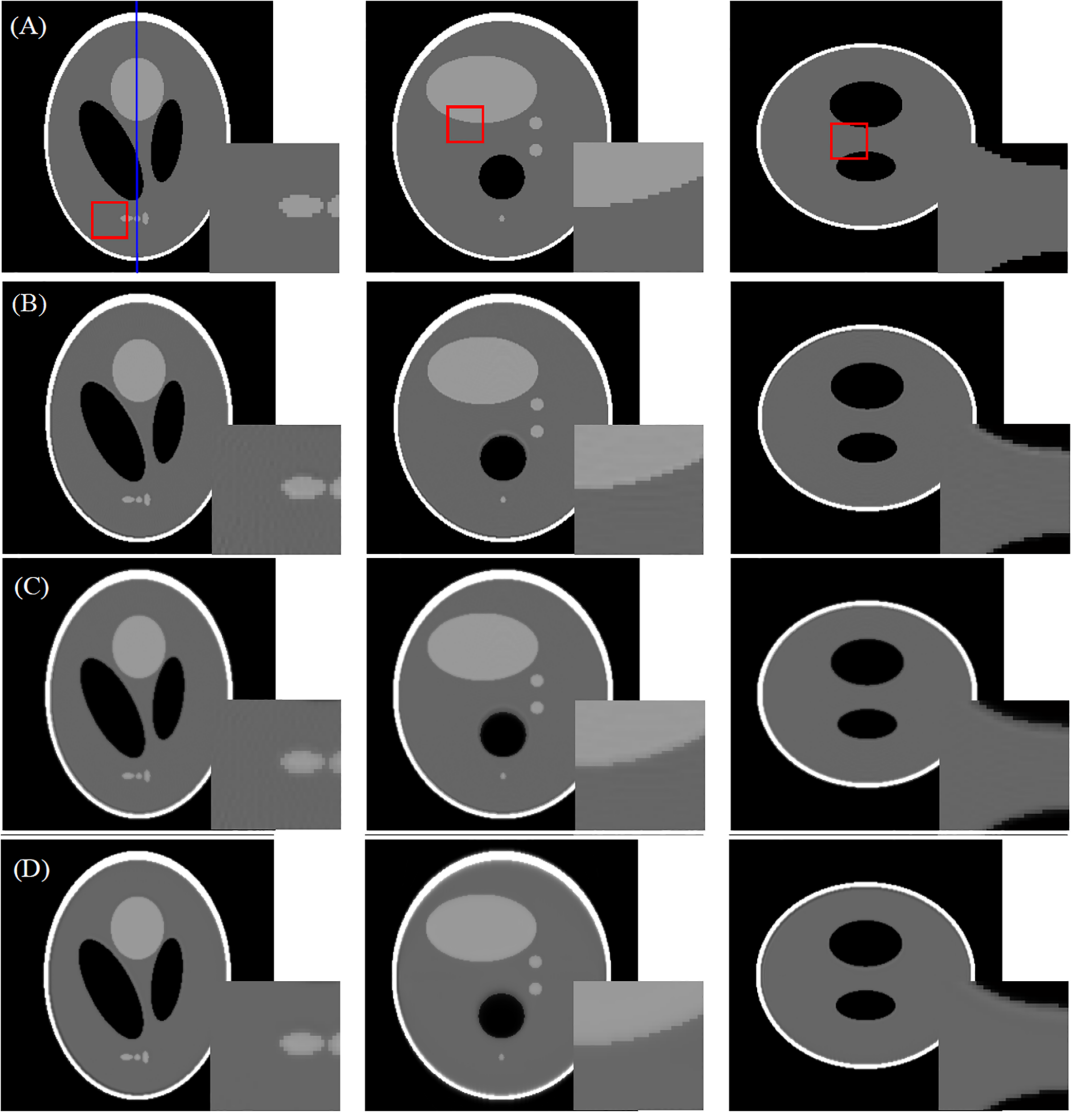
OSEM reconstructed results using different projection and back-projection operators. Columns from left to right are the illustrations of cross section, coronal section and sagittal section, respectively. Row (A): phantom images; Row (B): reconstruction with linear interpolation based FSNP and voxel-based back-projection (RMSE = 2.58%); Row (C): reconstruction with distance-driven projection and back-projection pairs (RMSE = 2.34%); Row (D): reconstruction with blob based FSNP and voxel-based back-projection (RMSE = 1.96%).

**Fig 6:**
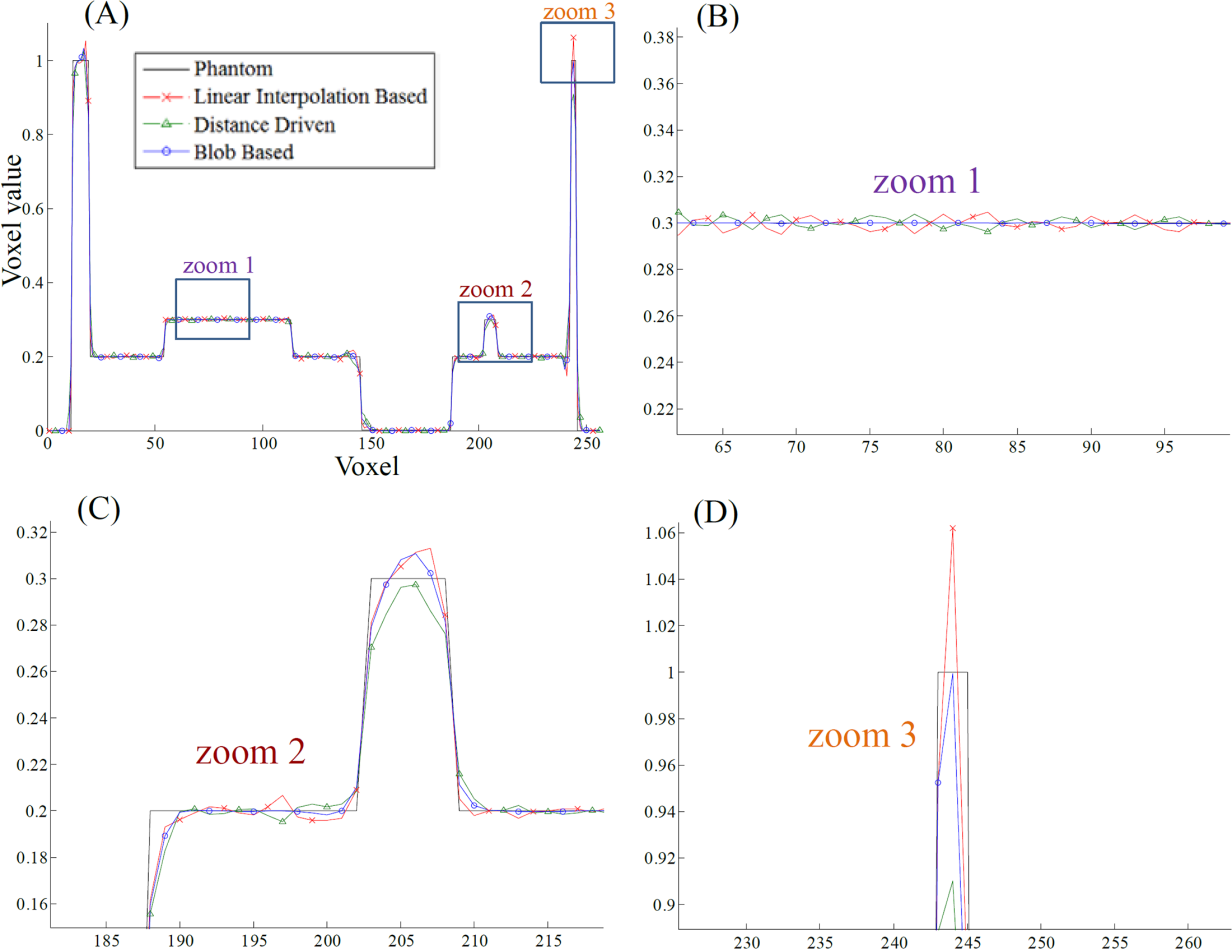
Profiles of the reconstructed images in Fig 5. The profile is marked in blue line in Fig 5(A), 5(B), 5(C) and 5(D) are the zoomed regions in marked as “zoom 1”, “zoom 2” and “zoom 3” respectively.

We then evaluated the performance of FSNP in iterative reconstruction algorithm. The OSEM (Ordered Subsets Expectation-Maximization) algorithm with 30 subsets and 100 iterations was chosen as the iterative algorithm. Results in Figs 5 and 6 show that the linear interpolation based FSNP together with voxel-driven back-projector can provide results similar to the matched distance-driven pair, both visually and quantitatively. Although the measurements are assumed to be independent form each other in ideal projection model, they are in fact somehow correlated, due to the imperfect collimation and scatter effect. The blob-based kernel takes such correlation into consideration, and thus leads to a more realistic model in characterizing the residual between observed measurements and image projections, especially for differences near the edges. As a result, we may see that different from the results in Fig 4(F), the reconstructed images in Fig 5(D) indicate that the blob based FSNP also leads to a good preservation of edges in addition to artifact suppression. Fig 6 plots the line profile (the vertical blue line in the left image in Fig 5(A)) of the reconstructions in Fig 5, from which we can see that the reconstruction with blob based FSNP results in a smoother profile with a better match of the reference profile than others (zoom 1); the distance driven projection provides blurred edges (zoom 2); and the blob based FSNP has the best performance in recovering peak value (with the best matched profile with phantom data in zoom 3).

**Fig 7:**
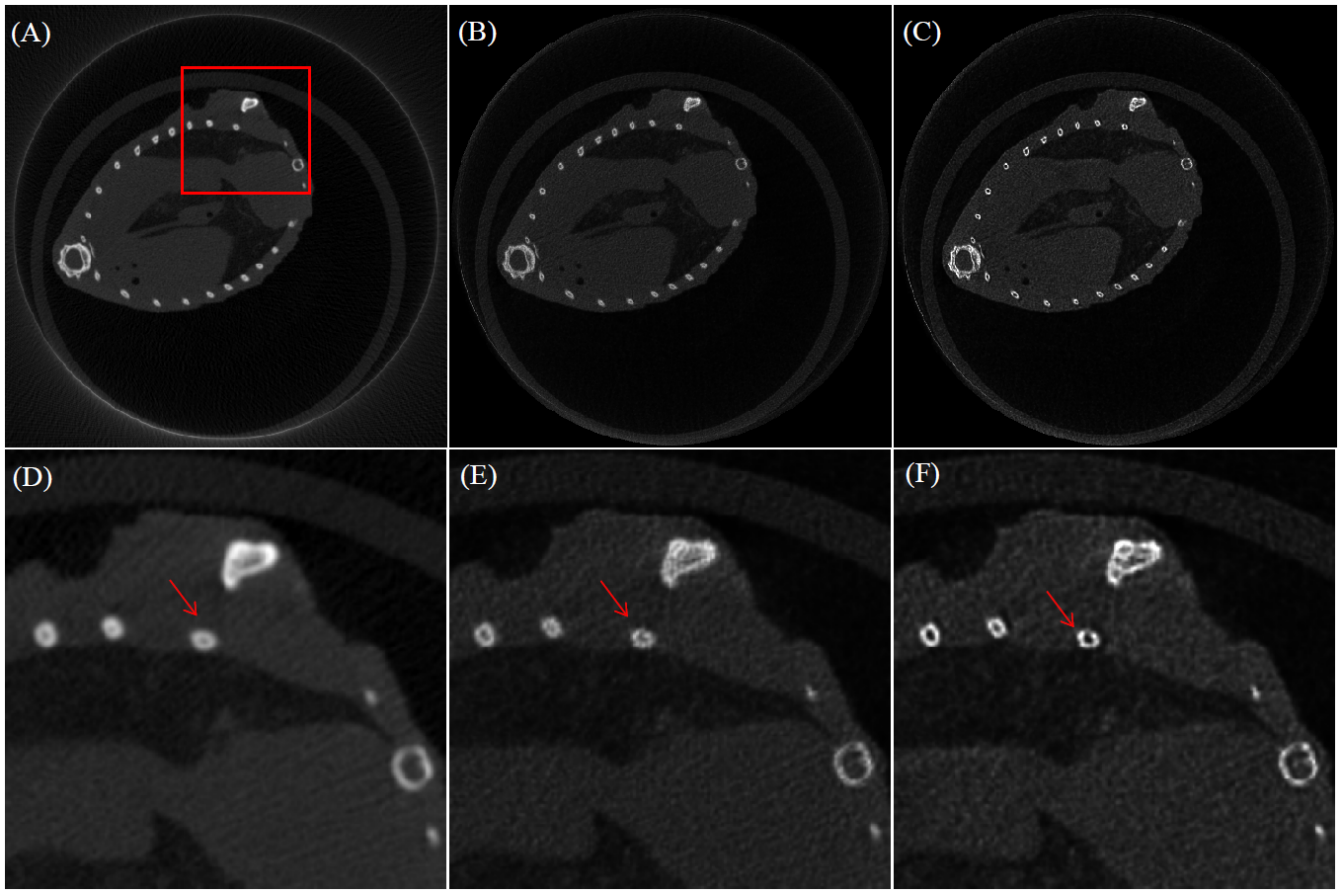
One 2D slice reconstructed from one rat data scanned from a micro CT. (A): FDK with Ram-Lak Filter; (B): OSEM with linear interpolation based FSNP; (C): OSEM with blob based FSNP. (D), (E) and (F) are the zoomed regions from (A), (B) and (C), respectively.

The proposed projection methods are also validated by real scan data from a micro CT system. A rat was scanned under 40kV tube voltage and 200mA tube current. The projection sequence contains 360 projections over 360°. The projection image size is 922×748 with pixel size 0.1*mm* ^2^; the reconstruction volume size is 512×512×512 with voxel size 0.085*mm* ^2^, *SOD* = 83.65*mm*, *SDD* = 167.3*mm*. The FDK algorithm with Ram-Lak filter and the OSEM algorithm with 30 subsets and 100 iterations were performed. All the methods listed are with voxel-driven back-projector. Different from the observation in Fig 5, the reconstruction results Fig 7 indicates that, compared to the linear interpolation based FSNP, the blob based FSNP provides results with better edge preservation (see the arrows). Nevertheless, we can also see that the improved edge preservation is also accompanied with amplified noise in the reconstructed images.

## Discussion

CUDA technology provides a well-established software platform for developers to design parallelized workflow with C-style code, with direct access to the virtual instruction set and GPU memories. In this paper, a strategy termed Fixed Sampling Number Projection (FSNP) is devised to ensure the operation synchronization along projection lines in parallelizing ray-driven projection based on CUDA framework. The conventional cubic FOV is replaced by a rotational symmetric FOV to save computation cost in geometry parameter calculation. Back-projection parallelization is relatively simple because the computation involved in each kernel for each back-projected voxel is independent to the computation in other threads. But as to projection operator, which calculates projection values via line integration of sample intensities, the involved accumulation operation often results in racing condition that limits the computation speed. Such problem can be alleviated by memory management and synchronization optimization.

Although there is a common view that PRPB mode is more efficient than PRPT mode in parallelizing CT projection using CUDA, experiment results have shown that with texture fetching, the computation time for PRPT mode is 43%~67% less than that of the PRPB mode. This advantage is brought by the locality property of texture memory when fixed sampling number is used for each ray. It is also found that the cost in texture binding occupies a large part in the whole computation. For instance, as to the PRPT mode with linear interpolation, in which the computational time for each projection in FSNP was respectively 17.09ms and 18.95ms for the cases with 256 and 512 sampling points per ray, there is 14ms cost for texture binding. For high resolution mode, the texture binding for 512×512×512 objects took about 104ms. Compared to the case with projection operator, the texture binding for back-projection requires less computation time due to the smaller 2-D data size (about 0.9ms and 2.8ms for 512×512 and 1024×1024 projection images, respectively). Despite this, the utilization of texture memory allows building a more accurate model by increasing the sampling points, without significantly increasing computational load.

Although fixing the sampling number in each line leads to more effective parallelization, it also results in inconsistent sampling density for projection rays, due to the different intersection lengths for projection rays within the FOV. For instance, the centered projection ray is often with a higher sampling density for cubic FOV; yet it is with a lower sampling density in the cases of cylinder or sphere FOVs. In the proposed approach, the sampling number is set based on the projection ray with the longest intersection length within the FOV, which defines the limit of the sampling intervals. Results in Fig 4 show that the MSE varies little when increasing the sampling number from 256 to 512.

## Conclusion

This paper proposes an effective parallelization scheme FSNP for the projection in iterative CT reconstruction algorithms. In this FSNP method, the sampling point number on each projection ray is fixed to ensure the synchronization of parallel computing. Texture memory is also used in the FSNP approach to further improve the computation efficiency. Experiment results show that the proposed approach with texture memory is 10~16 times faster than the global memory version in iterative reconstruction. Although it is widely accepted that the PRPB mode is suitable for the parallelization of the projection operator, this study indicates that the PRPT mode is about twice faster than PRPB mode with the proposed approach. We also found that the proposed ray-driven base FSNP method works well with voxel based back-projection operator in reconstructions. By introducing blob based kernel functions into the FSNP method, better reconstruction results than the distance-driven projection and back-projection pair can be obtained by the proposed approach. Better performance in noise suppression can be expected to be obtained by incorporating the noise property into kernel building.
